# Practical Departments and New Appliances

**Published:** 1905-06-17

**Authors:** 


### PRACTICAL DEPARTMENTS AND NEW APPLIANCES.

### SANITARY FITTINGS AT THE KING'S SANATORIUM.

### The Operating Theatre.

For the operating theatre two strong white-glazed fireclay sinks with rolled edge have been selected, glazed inside and out with recess for gun-metal removable standing waste, and supported on vitreous enamelled cast-iron cantilevers to stand clear of wall. Each sink is fitted with Doulton's patent gun-metal, non-scalding valves, with lever handle to be actuated by elbow, one fitting to have a spray, and the other an anti-splash jet; between the sinks a polished marmorite slab will be placed supported on polished gun-metal brackets with removable clips, so that the slab may be removed for cleaning. In addition to the sinks two strong white earthenware lavatories will be provided fitted with wastes and valves of the same kind as the sinks. In every case fittings are rounded off at all angles, all exterior surfaces glazed, and everything open for inspection and cleaning. Floor-channels are largely used for carrying off the waste water, each channel having a strong hinged dome grating at the outlet, and a glazed fire-clay trap.

### The Lavatories.

The lavatories are strong white glazed fireclay, with gun-metal standing wastes, and valves made with bibs to stand sufficiently high above the pottery to allow of proper cleaning, they are carried on white vitreous enamelled cantilevers, and kept clear of the wall. The sinks are of various sizes, and have rolled edges in front and at the sides, but the backs are square so that the wall tiling will rest on them; they are glazed both inside and out. Some of the sinks have oiled teak tops instead of the rolled edge, and are fitted with gun-metal removable standing wastes. The bed-pan and scalding-sinks are of special design, and include a bed-pan sink, a 4 feet long scalding-sink and drainer, all being glazed both in and out. The bed-pan sink is fitted with a rising jet for bed-pans and a cleanser for urine bottles, the jets are flushed by screw-down valves and are connected to both hot and cold supply, and the bed-pan is flushed by a syphon cistern. Two special sinks are provided for the service-room and pathological post-mortem room; these are of large size, glazed all over with removable standing waste and gun-metal ¾-inch hot and cold valves; they are supported on cantilevers.

### The Closets.

The closets are made in strong white glazed fireclay of the wash down type and the back is carried right to the wall with a slight skirting so that no space is left that will in any way conceal dirt. The closet is carried on two cantilevers built into the wall, and these are fitted into a pottery slieve so that no part is exposed, the cantilevers keep the closet well clear of the floor, there is, therefore, no part that cannot be cleaned. The cisterns and covers are cast iron white vitreous enamelled, and made perfectly plain with all angles well rounded, and placed on special brackets which keep the tanks clear of the wall. The seats are oiled teak made in two thicknesses with the grain crossed to prevent warping. Two closets are fitted with a special swing jet to be used for cleaning bed pans, this is screwed to the wall, and can be swung clear of the wall when not required. The patients' baths are white, glazed inside and out, and arranged for the outlet to discharge into a channel fixed below the floor level. The supply valve is a patent fitting of Messrs. Doulton's, it is arranged for the water to come through one bib, and the temperature can be regulated as desired, but the one most important point is, that the hot water cannot possibly be turned on first, and, being worked by a loose key—a feature that will appeal to all interested in hospital or asylum work. The staff baths are made in cast-iron, white vitreous enamelled inside and out, and have a combined removable standing waste and overflow fitted in a recess, with one-inch valves. The whole of the sanitary fittings are being supplied by Messrs. Doulton.

### FIRE-EXTINGUISHING APPLIANCES.

### The Grinnell Sprinkler.

The Grinnell Sprinkler, made by Messrs. Mather and Platt, of Salford, Manchester, is an automatic appliance, intended to deluge an area surrounding each sprinkler of 100 square feet floor space. The building to be protected has pipes fixed in every room, from 8 to 10 feet apart, and to the pipes are attached sprinklers, also 8 to 10 feet apart. The pipes are connected to any convenient source of water-supply. The sprinkler itself consists of a valve held in an annular disc attached to a short pipe, the latter screwing into its place in the service-pipe mentioned above. The valve is a smooth glass hemisphere resting upon a diaphragm of German silver, the circumference of the glass valve filling a circular hole in the diaphragm, while it is in its place, and so preventing the egress of the water with which the pipes are full. When the glass valve is lifted off its seat in the diaphragm, water can pass through. The valve is kept in its place, partly by the tension of the German silver diaphragm pressing it upwards, and partly by what the makers call a strut fixed usually below it. The strut consists of three pieces of metal, held together by soft, low temperature fusing solder, and it holds the valve to a metal arch projecting from the disc in which the valve and diaphragm are held, exerting a pushing force, to keep the valve on its seat, as against the effort of the diaphragm, and the water in the pipes to push it out of its seat. The metal arch which supports the strut also carries a special form of rose, or sprinkler, consisting of a notched disc. If the temperature in the neighbourhood of any sprinkler reaches 155° F., the solder holding the strut together melts, the glass valve moves from its seat, and a stream of water passes out through the valve and is broken up and spread by the rose at the top, very much as the rose of a watering-can does, but with larger streams. The stream of water continues as long as the supply lasts, or till the valve is replaced. Where the water in the pipes will be liable to freeze, air under compression is used to open a valve allowing the water to pass into the pipe where the sprinkler is open, the latter opening by the solder of the strut melting, as before. Connected to the Grinnell system is an automatic alarm, which gives warning in the engine-house, or wherever may be desired, not only of a sprinkler being open, but of leakage in the pipes, so that the system is always maintained automatically in working order. The pipes for the sprinkler are fixed usually in the ceiling. About 50,000 buildings in all parts of the world are stated to use the Grinnell sprinkler, 10,000,000 being fixed in the 50,000 buildings.

### HEATING AND COOKING APPARATUS.

### Messrs. George Wright and Co.'s Specialities.

Messrs. George Wright and Co., of 155 Queen Victoria Street, London, E.C., have made a speciality of heating and cooking apparatus for hospitals, and a very large assortment of their different manufactures may be seen at their show rooms. In particular we noticed the large handsome cooking ward-stoves, arranged to be fixed, either in the centre or at the ends of the wards, with flues above the stoves or below as convenient. In the latter case, the flue is taken down into the floor, and thence to the chimney by a horizontal flue in the floor. The stoves themselves are made in various designs, including the well-known pattern in which the outside of the stove is covered with ornamental glazed tiles, ornamental iron work, and others. The air of the ward is warmed in two ways by these stoves; there is the ordinary cheerful fire-place with its glowing coals, sending out heat rays into the ward, and in addition, there is a supply of air from outside constantly passing into the ward warmed as it passes through the stove. Air is led from a grating in the outer wall, to a space at the back of the fire surrounding the flue, and thence into the ward by adjustable outlets in the front of the stove. The air is exposed to the heat of the back of the fire and of the flue in its passage from outside into the ward, and its temperature is raised. There is no possibility of the smoke from the flue mixing with the air that is passing into the ward round the flue, as long as the stove is in order. Special arrangements are made in the construction of the stove for cleaning the air passages, and this applies also to the downward flues. The arrangement for getting at the air passages for cleaning the stoves is by simply removing the outlet ventilator in front and a movable panel at the side of the stove, the whole space being then easily reached with a flue brush. The air spaces in the large ward-stoves have baffles or gills fitted at different parts of the air-course to increase the heating surface, and to delay the air, so that it may absorb more heat in its passage. Messrs. George Wright and Co., make a number of other forms of stoves, on very much the same lines, but for fixing against the wall, in the usual position, and for day-rooms, nurses' rooms, etc. Some of the stoves have firebrick backs, and some again have special arrangements, rendering them suitable for lunatic asylums. The firm also make a number of cooking stoves of various patterns, on the same lines as those described in connection with ward stoves, as fire ranges for the middle of the kitchen, with descending flues, carving tables, and hot closets in one apparatus, the top plates being warmed by steam passing in a hollow space under the plates and the closet by a separate supply of steam. They make the usual steam appliances for cooking vegetables, boiling soups, etc.

### RADIANT ANTHRACITE COAL STOVES.

In this country anthracite coal has hardly received the attention it deserves, partly on account of its high price, and partly because of the difficulty experienced in burning it. Anthracite is the last product in the coal series. Coal is formed from vegetation which grew ages ago, and which has been subject to pressure, and chemical and other physical action, passing through the various stages of peat, brown coal, bituminous, semibituminous, and finally anthracite, in which form it is nearly all pure carbon, and has a very high calorific value. The great heat which anthracite gives out has proved somewhat of a stumbling block in the way of its adoption for domestic and similar use. Mr. E. E. Pither, of 36 and 38 Mortimer Street, London, W., who has carefully studied the subject, both theoretically and practically, has solved the problem, he claims, in such a manner that the cost of heating rooms, such as the wards of hospitals, of small size, is effected more economically and more conveniently than with the ordinary fire-grate, burning the ordinary house coal. The "Radiant" stove burns for 36 hours without any attention, with one charge of coal, at a cost of one penny for six hours. The stove itself is practically a sealed coal hopper, with a specially-arranged fire-grate attached. It is usually cylindrical in form, constructed in iron, with a grate at the bottom, having front bars which turn on horizontal axes, a movable bottom to the grate, and an ash pan below it. At the back is the flue, with a regulator to control the draught, and about half-way to the back of the grate is a fire-brick, filling the place, roughly, of the bridge in the furnace of a steam boiler. A special shovel is supplied with the stove, consisting of a plate of metal with a handle, the shovel being intended to be forced between the front bars of the grate. To start the stove the shovel is placed between two of the bars in a groove provided for it near the top, and the anthracite coal is filled into the stove down to the shovel. The cover of the stove, which has a silver-sand seal, the rim of the cover fitting into a groove filled with sand, is then put in its place, and the fire lighted below the shovel with ordinary bituminous coal. When the fire is well alight the shovel is withdrawn, and the anthracite allowed to descend on the lighted mass, where it ignites. As it consumes the coal in the stove gradually descends, the fire presenting a cheerful appearance through the front grate bars, and giving out considerable heat. To liven the fire at any time, the shovel is inserted at the first or second groove from the bottom, to hold the fire above it up, the lower movable part of the grate is then pulled out by means of a hook provided for the purpose, its contents tipped into the ashpan, and the movable part replaced, the shovel being then withdrawn, the coal settling down and making a fresh fire. The "Radiant" stove is made in sizes from 9 in. diameter, by 2 ft. 7 in. high, suitable for rooms 12 ft. by 10 ft. high, up to 19 in. diameter, by 3 ft. 9 in. high, suitable for rooms of 20,000 cubic feet area. The stoves are also frequently combined with boilers, the water from which may be used for domestic purposes, or for heating radiators of the ordinary pattern. In one form the boiler stands at the back of the stove, surrounding the flue, and in another the boiler is divided into two, standing on the two sides, forming ornamental extensions. We understand that Mr. Pither has recently fitted the waiting-room of the Eye and Ear Hospital at Tunbridge Wells with one of his Radiant stoves, the operating-room above being also warmed by a radiator using the water heated in the boiler attached to the stove. The "Radiant" stove can be fitted to any fire-place, or anywhere from which a flue can be carried to the chimney.

### THE PULSOMETER ENGINEERING COMPANY'S COLD STORAGE APPARATUS.

The Pulsometer Engineering Company of Nine Elms Works, Reading, are known best by the beautiful pumping apparatus they have been instrumental in putting on the market, but they are also general engineers, and amongst other apparatus they have made a speciality of cold storage plant. Their apparatus is made on the ammonia compression system, as described in the articles on the Engineering Side of Hospital Work, and their claims are therefore not for orginality so much as for good workmanship. For brine circulation, calcium chloride is used, as is general. All the ammonia fittings are made of wrought iron and steel, the firm claiming that it is not possible to secure immunity from leakage, with its unpleasant, and possibly disastrous consequences, with cast iron, and they have put down special machinery to deal with the fittings, so as to make them at reasonable cost. The firm claim that by their system of fitting the gland to the ammonia compressor, and the headers for connecting the successive lengths of pipes forming the condensers and expansion coils, the possibility of leakage of ammonia is reduced to a minimum. It will be remembered that, in the compressor, the ammonia gas is under considerable pressure, and is raised to a high temperature, and the tendency for the ammonia to escape through imperfect joints is great. Different manufacturers have different methods of overcoming the difficulty, the Pulsometer Company's being to use only wrought iron and steel, and to oppose metal, very accurately fitted, to metal, the surfaces in contact and the fitting accomplishing all that is required. The company make all sizes of ammonia compression plant. Among those suitable for hospital use may be mentioned a 1½-ton combined plant, with engine, condenser, and evaporator on one bedplate, the condenser being separate from the evaporator; a 10 cwt. self-contained plant on somewhat similar lines, and others. The apparatus named can be driven by electric motors, or by belts from any convenient shafting, instead of by separate engine, if desired. They also make a special plant for tropical climates, consisting of a horizontal engine directly connected to the ammonia compressor, which is carried on the same bedplate, with the condenser mounted on a brick foundation alongside, the brine pump being driven by the engine shaft, and the evaporator coils and brine coils arranged as convenient.

### A SEWAGE PLANT FOR BUILDINGS AWAY FROM LARGE DRAINAGE SYSTEMS.

The problem of dealing with the sewage of buildings away in the country, far from any large system of drainage, is often one of considerable difficulty. In the old days the cesspool or dumb well was employed, but in these days the sanitary authorities would hardly allow such a thing, while the greater enlightenment upon all these matters condemns them. Mr. William Farrer, of the Star Works, Cambridge Street, Birmingham, has stepped in to help the administrators of hospitals, convalescent homes, and similar buildings, situated as described, and his apparatus is designed upon the lines that have been accepted, up to the present, as those best able to deal with sewage, namely, the submission of the sewage to the action of anærobic bacteria for a certain time, and to ærobic bacteria for a further time. In Mr. Farrer's apparatus the sewage is all run into a tank provided for its reception, which is closed to the ingress of both light and air, the settling, or liquefying, or septic tank, as it is variously termed, where it is subjected to the action of the ærobic bacteria. When the level of the sewage in the tank reaches a certain point, it is allowed to pass out to the ærobic treatment by a tube situated beneath the surface of the sewage. The sewage passes from this tube into a tippler, or tumbler, the object of which is to automatically deliver the sewage to half the ærobic filter-beds in succession. The tippler consists of a vessel divided into two halves, each of which, when horizontal, holds the sewage passing into it, but, when weighted with a certain quantity, tips over and allows the sewage to pass to the distributor on the other side of the tippler, the other vessel then receiving the sewage, turning over in its turn, and so on. From the tippler the sewage passes to a number of troughs arranged to receive it, the troughs being carried a little above the filter-beds and being perforated on the under sides with small holes, so that as the sewage passes along it is sprayed out through the fine holes, on to the filter-bed, even distribution being thus obtained. The ærobic portion of the apparatus consists of a second tank, filled with a filtering medium. Mr. Farrer uses "saggers," the refuse from a certain portion of work in the potteries, crushed to convenient sizes and graded, the smallest being at the top, and larger and coarser for the deeper portions. The bottom of the filter has a false floor, formed by perforated tiles of the interlocking pattern. The bottom of the filter is of concrete, with channels for the liquid to run out. The false bottom allows air to pass under the filtering medium, and assists to keep the floor clean. The effluent, the liquid finally obtained after the sewage has passed through both tanks, passes into the soil in a condition, it is claimed, perfectly harmless to vegetable or animal life. The effluent is stated to be good for treating gardens with, as it is rich in nitrates. The maker claims great simplicity for his apparatus, and that by the spraying arrangement the filter bed is treated in the best possible manner, and is never flooded. The apparatus is applicable to buildings having sewage up to 10,000 gallons a day to dispose of. This means, in the country districts to which the apparatus is specially suitable, buildings where 500 persons are housed, including nurses, patients, and other employés. The settling tank has a small "sump" in the centre of the bottom, into which substances that are not capable of being liquefied can fall. Right above the sump is an air-tight man-hole door that can be lifted off occasionally for the purpose of cleaning out the sump. Mr. Farrer has fixed, among others, sewage plant on the above system at the Derbyshire County Asylum, Mickleover, where the filter bed measures 30 feet by 15 feet, and at the West Stoneham Union, near Southampton, where two filter beds, each 30 feet by 20 feet, deal with 20,000 gallons of sewage per day.

### A PORTABLE GAS-HEATED RADIATOR.

In the duplex radiator, which comes to us from America, a distinctly new line has been struck. The apparatus is thoroughly portable, just as an electrical heating stove is, the flexible metallic tube which connects the radiator to the gas service not being very much heavier than a properly designed cable for an electric radiator, and there is no chimney, it being claimed by the makers that there is no smell nor deleterious substance given off from the radiator to a sufficient amount to require carrying to the chimney. The apparatus is also constructed on entirely novel lines, so far as the consumption of the gas used for heating is concerned. The usual method of burning gas for heating purposes, as is well known, is to mix a large proportion of air with the gas at the burner, the gas then burning with a blue flame, on what is called the Bunsen principle, from its distinguished discoverer. Everyone is familiar with the fact that the ordinary fish tail and bats-wing burners are very wasteful in gas, and that they do not give out as much light or heat for the same expenditure of gas as the Welsbach mantle, because the particles of carbon are cooled so quickly in their passage outwards from the centre of the flame, and because the Welsbach apparatus is really a Bunsen burner, which generates sufficient heat to raise the mantle to incandescence. Nevertheless the materials for the generation of heat are present in the fish-tail burner, if they are properly utilised, and this is what the inventor of the duplex radiator claims to have done. The apparatus consists of pairs of fish-tail burners, four pairs for the four-tube and six pairs for the six-tube, the burners being carried by a pipe fixed in a hollow casting forming the base of the apparatus, standing slightly off the floor, so that air can pass in underneath. Above each pair of burners is a tube of thin sheet iron, while above the tubes again is a second casting, shaped like an inverted dish. The tubes are held between the base and the upper casting by rods passing through the base and the top plate, and the tubes are held in position by slipping over rings cast on the base, and by wedge-shaped spikes passing into the tops of the tubes from the top casting. But the wedge-shaped spikes are only allowed to enter the tubes to a certain depth, so that there is a small space left between the tops of the tubes, and the under side of the upper casting, the latter curving round over the tops of the tubes so as to conceal the space left free. In the upper portion of the tubes a number of discs with deeply serrated edges are suspended, the points of the disc plates touching the inner surfaces of the tubes, and the spaces formed by the serrations allowing the passage of the products of combustion. The working of the apparatus is as follows :—The usual combustion of gas goes on at the fish-tail burners, but the products of combustion pass up the tubes, and instead of passing freely into the atmosphere, are checked by the plates in the upper portion of the tubes; the space between the burners and the lowest plate becomes very hot, 450° F. it is stated. The result is that the half-burned products of combustion from the fish-tail burners are exposed to this heat, and are thoroughly burnt, giving off more heat in the process, this being radiated into the room from the outer surfaces of the tubes. The final products of combustion pass up between the serrated plates and the inner surfaces of the tubes, and out to the atmosphere by the space left free under the dish-shaped top plate. The air which feeds the burners is also heated by passing through the heated base, the latter being raised to a considerable temperature by contact with the tubes and by the heat given off from the burners. The top plate does not become very hot, a peculiarity of the apparatus due evidently to the passage of the air through the space mentioned, between the tops of the tubes and the top plate, and to the latter only being connected to the tubes by the wedge-shaped spikes. It is stated that a consumption of from 10 to 12 cubic feet an hour is sufficient for heating rooms of the usual size of doctors' consulting rooms at hospitals, and it should therefore be very useful for that purpose on account of its portability. It is stated to be already in use in many private consulting rooms. The address of the Duplex Radiator Company is 37A Southampton Row, London, W.C.

### A LOW PRESSURE STEAM HEATING APPARATUS.

In the steam heating apparatus that is supplied by Messrs. Korting Bros., of 53 Victoria Street, London, S.W., in which the steam-pressure, at any part of the service, never exceeds 1½ lb. per square inch above that of the atmosphere, it is claimed that the advantages of heating by steam and those of heating by hot water are combined. With hot water, it will be remembered, the heat given out at any radiator can never be above the boiling point of water, and in practice is always considerably below it, but the pipes and radiators have to be made larger than where steam is employed to furnish the same result. The heat of live steam above atmospheric pressure is always above that of the boiling point of water, and considerably above that of the water usually employed in heating apparatus, and it is claimed by advocates of hot water heating apparatus that inconveniences result, such as the burning of dust in the air which impinges upon the hot surfaces of the radiators. Hence, for hospital work, hot water radiators have been almost universally employed. On the other hand, heating by steam is claimed by the advocates of steam heating apparatus to be very much more economical where it can be applied. In the radiators supplied by Messrs. Korting Bros. the difficulty is met by using the low-pressure steam mentioned, and delivering it to the radiators in such a manner that the heat delivered by the steam is spread over the whole surface of the radiator, and the actual temperature of the surfaces of the radiators is less than that of the steam by an appreciable amount, the temperature approaching in fact to that of hot-water radiators. This object is attained by delivering the steam to each individual section of the radiator through a nozzle, that is, in a very fine jet, the result being that the steam passes from the bottom of the radiator, where the supply-pipe is fixed, right up to the top, mixing with the air in the radiator and so becoming lowered in temperature. There are no air-valves attached to the radiator, any air that may be present that is not required to take part in the above action being carried off with the water formed by the condensing steam to a vessel provided for it or ejected to the atmosphere. It is claimed also that the temperature is kept completely under control without the use of thermostats by the steam entry-valves at the radiators and by an automatic apparatus at the boiler which controls the air-supply to the boiler furnace.

There is, unfortunately, not space in this notice to describe fully the beautiful arrangement by which it is attempted to accomplish this object. Briefly, the supply of air to the boiler-furnace is controlled by a door at the side of the furnace, which is opened to a greater or less extent according to the requirements of the service; and the door is moved indirectly by the steam-pressure in the service. The pressure at which the service is to be worked, can be varied from 1½ lb. per square inch, down to ¼ lb. per square inch, above the atmosphere, by moving a weight along a lever which forms part of the control of the air-supply door of the furnace. Once the pressure is arranged, the supply of air is controlled by the steam-pressure in the boiler. Thus, if steam is taken away rapidly by the radiators, the pressure in the boiler is lowered, and this acting through the medium of a column of water, two floats and some levers, opens the air-door. If, on the other hand, the service is taking very little steam, the same arrangement pushes the air-door closer to its seat. In extreme cases, where the pressure rises very rapidly from any cause, the air-door is quite closed, and cold air is admitted by the same system of floats and levers, to the boiler flues, effectually checking the generation of steam by the hot gases from the furnace. Precautions are also taken, automatically, for ensuring that the boiler cannot be worked with a dangerously small supply of water, the steam generated under such special conditions being condensed and returned to the boiler. It is further claimed that there is very little chemical action upon the internal surface of the pipes of the service, as the oxygen available is used up soon after the apparatus is put into service. This should have an important bearing upon the efficiency of the apparatus, as oxide of iron is a poor thermal conductor.

### AN ELECTRICAL APPARATUS FOR KEEPING FOOD WARM.

Messrs. Duncan Watson and Co., of 100 Charing Cross Road, London, W.C., have recently constructed a novel arrangement for keeping dinners, etc., warm in transmission between the kitchen and the wards. It consists of a small trolley, fixed on rubber wheels, arranged to run on the lift.

On the trolley are several shelves, on which dishes can be placed, and at the bottom is a hot plate warmed by electricity. The hot plate consists of a thin steel-topped box, in which an electrical resistance is coiled, the resistance usually being a German silver or managanin strip insulated with asbestos. When an electric current passes through the metal strip it becomes hot, and the steel plate on the top gives out heat to the dinners above. The ends of the metal strip are connected to a flexible cord with a double plug at the end. When the trolley is standing outside the kitchen waiting for its load the flexible cord is plugged to a corresponding service-plug, arranged near, and the steel plate is heated up. When the trolley is taken to the lift the plug is taken out of the service-plug, the flexible cord coiled up, and the trolley run on the lift. If there is an electric service on the lift it is an easy matter to arrange a service-plug on the lift in connection with it. In any case, when the trolley is run off the lift it is again connected to the electrical service by means of service-plugs arranged for the purpose in the corridors, wards, etc.

### HALL'S SANITARY DISTEMPER.

(163 Queen Victoria Street, E.C.)

Hall's Sanitary Distemper enables cleanliness and smartness to be secured with ease and economy. Wherever there is a handy man it is applicable without the offices of the painter and decorator. Indeed, so easy is it of application, that in small buildings the handy woman also is quite equal to its use. It is prepared both for out and indoor painting, and will be found very excellent for either. It is supplied in seventy shades of colour.

